# Collecting big data with small screens: Group tests of children’s cognition with touchscreen tablets are reliable and valid

**DOI:** 10.3758/s13428-020-01503-3

**Published:** 2020-12-02

**Authors:** Giacomo Bignardi, Edwin S. Dalmaijer, Alexander Anwyl-Irvine, Duncan E. Astle

**Affiliations:** MRC Cognition and Brain Sciences Unit, University of Cambridge, 15 Chaucer Rd, Cambridge, CB2 7EF UK

**Keywords:** Cognition, Reliability, Validity, Childhood, Tablet, Group testing

## Abstract

Collecting experimental cognitive data with young children usually requires undertaking one-on-one assessments, which can be both expensive and time-consuming. In addition, there is increasing acknowledgement of the importance of collecting larger samples for improving statistical power Button et al. (*Nature Reviews Neuroscience 14*(5), 365–376, 2013), and reproducing exploratory findings Open Science Collaboration (*Science*, *349*(6251), aac4716–aac4716 2015). One way both of these goals can be achieved more easily, even with a small team of researchers, is to utilize group testing. In this paper, we evaluate the results from a novel tablet application developed for the Resilience in Education and Development (RED) Study. The RED-app includes 12 cognitive tasks designed for groups of children aged 7 to 13 to independently complete during a 1-h school lesson. The quality of the data collected was high despite the lack of one-on-one engagement with participants. Most outcomes from the tablet showed moderate or high reliability, estimated using internal consistency metrics. Tablet-measured cognitive abilities also explained more than 50% of variance in teacher-rated academic achievement. Overall, the results suggest that tablet-based, group cognitive assessments of children are an efficient, reliable, and valid method of collecting the large datasets that modern psychology requires. We have open-sourced the scripts and materials used to make the application, so that they can be adapted and used by others.

Traditional one-to-one cognitive and behavioral assessments remain the gold standard in most aspects of psychological assessment. However, many standardized tools can be costly to purchase, and time-consuming to administer and score. This constrains the possible sample size of most studies, which is increasingly at odds with using innovative new methods (e.g., machine learning) that require larger sample sizes, and against recent moves to ensure that psychological science is robust and replicable (Button et al., 2013; Munafò et al., 2017). Additionally, relying solely on one-to-one testing imposes other limitations that may be more specific to the sample being studied. For example, research with young children frequently utilizes small convenience samples of families with the time and interest to attend a lengthy one-to-one assessment in a research lab, jeopardizing external validity (Keiding & Louis, 2016).

For research that requires detailed cognitive performance metrics with school-aged children, one alternative data-collection approach is to test groups of children in schools. This approach may reduce obstacles to research participation, which is especially important for recruiting groups that are typically under-represented in research, such as low socioeconomic status families (Jacobsen, Nohr, & Frydenberg, 2010; Sakshaug, Schmucker, Kreuter, Couper, & Singer, 2016; Winding, Andersen, Labriola, & Nohr, 2014).

Despite the widespread adoption of group testing in education (e.g., SATs, GRE), it has been less widely adopted in developmental research. This might be explained by concerns about reliability, distractibility, and low motivation during group tests (Gregory, 2014; Murphy & Davidshofer, 2004). Auditory distractions such as acoustic noise can impact task performance in children (Joseph, Hughes, Sörqvist, & Marsh, 2018; Röer, Bell, Körner, & Buchner, 2018). However, classroom noise may not impact test reliability nor decrease inter-task correlations (Kanerva et al., 2019).

When adapted well, group tests can have excellent psychometric properties. For example, the widely used assessment tool WAIS-R has been adapted into a pencil-and-paper group test for ages 16+, the Multidimensional Aptitude Battery (MAB-II). MAB-II test–retest reliabilities and task correlations to individually tested WAIS counterparts are high (Harrell, Honaker, Hetu, & Oberwager, 1987; Jackson, 1998; Luciano et al., 2003). For young children, the well-validated CAT-4 pencil-and-paper group assessment covers quantitative, nonverbal, and spatial reasoning domains, also reports a high test–retest reliability (GL Assessment, 2012). However apart from a few exceptions (e.g., Brankaer, Ghesquière, & De Smedt, 2017), many popular assessments are yet to be adapted for group testing.

Whilst pencil-and-paper assessments may be the simplest tests to create for groups, they are limited in the types of tasks that can be administered, and the types of outcomes that can be measured (e.g., no reaction times or dynamic measures), and require laborious manual scoring. Many of these limitations are addressed by using computerized tasks. There may be specific benefits for using computerized tasks, over and above their paper-and-pencil counterparts. For example, they permit audio instructions and reminders, reducing dependence on reading skills. Existing studies have not found large differences in performance in pencil-and-paper compared to computerized versions of common tasks (Piatt, Coret, Choi, Volden, & Bisanz, 2016; Robinson & Brewer, 2016). But computerized assessments can come with multiple technical challenges of their own. For example, relying on a school’s IT system for administering tests can be hampered by bandwidth and browser issues (e.g.,Wassenaar et al., 2019).

Touch-screen tablet computers are another option for administering group cognitive tests and benefit from being relatively inexpensive and highly portable. The touch-screen interface of tablets is also easy to use: even children aged 2–4 can accurately complete basic tasks (Azah, Aziz, Syuhada, & Sin, 2014; Semmelmann et al., 2016). Preliminary evidence suggests that young children prefer tablet assessments relative to pencil-and-paper tests (Piatt et al., 2016). Automated, computerized tasks are also less likely to suffer from experimenter bias and errors in scoring and administration (Chapman, Benedict, & Schiöth, 2018; Styck & Walsh, 2016). Self-guided tests generally require less training to administer, and can thus be more easily implemented in large cohort studies (Bhavnani et al., 2019). However, few tools currently exist specifically designed for testing children in groups, and little psychometric work exists validating these assessments.

Well-established tablet applications for child cognitive testing exist, however they are specifically designed for a researcher to provide instructions and closely monitor adherence. The NIH Toolbox is designed for testing cognition across the lifespan, initially tested in a large sample aged between 3 and 85 years (Weintraub et al., 2013), and has since been incorporated into other large research cohorts (Akshoomoff et al., 2014; Thompson et al., 2019). For children aged 2 to 5, the Early Years Toolbox contains five cognitive tests with high internal consistency (Howard & Melhuish, 2017).

Research on tablet applications specifically designed and validated for group-testing children’s cognitive abilities, has been relatively mixed. Pitchford & Outhwaite (2016) tested a sample of Malawi and UK school children, aged 4 to 12 on seven cognitive assessments including short-term memory, working memory, and mathematics ability. Reported task reliability varied between 5% (working memory) to 73% (mathematics). The varying reliabilities of tasks highlight the importance of task design and highlight the importance of validation of novel measures. In contrast, Kanerva et al. (2019) report on two novel tablet-based working memory tasks in a large sample of 12-year-olds, finding a moderate correlation between tests (*r* = .44) and correlations to school grades (*r* = .42 & *r* = .36).

## Study overview

The present study evaluated the effectiveness of using self-guided tablet-based cognitive assessments to collect large-scale datasets, using group testing in school classrooms. While the code and materials used to generate the application are publicly shared and freely available for researchers to use, our primary goal here was to evaluate the general approach. The cognitive assessment was created as part of a large longitudinal study of children aged 7–9 years. The broader study aimed to collect a large, demographically representative dataset comprising behavioral, educational, cognitive, mental health, demographic, home environment, and teacher-rated measures. One group of children completed the assessments in groups in classrooms, and a smaller second group completed the same assessments individually in a laboratory setting, along with standardized cognitive tests (WASI-II).

We aimed to include a broad set of assessments in the application, which were divided into core and supplementary tasks. Core tests were chosen to tap key domains of cognition that have been highlighted as important for learning and school progress (Holmes, Bryant, & Gathercole, 2019), and could also be easily adapted for tablet use. These included: reading and arithmetic fluency, short-term memory, matrix reasoning, visual search speed, and number discrimination. We note that this list is not exhaustive. The supplementary tests were more novel, and included at the end of the battery, so not all children may have completed these if testing time ran short. Evaluation of the tablet assessments is divided in three sections: reliability, predictive validity, and measurement invariance.

### Reliability

Our first goal is to determine the reliability of the cognitive tasks. No psychological construct can be measured perfectly without measurement error. Reliability coefficients specify the proportion of variance in an outcome that can be attributed to “true” differences between individuals, as a proportion of total variance (true and error variance; Revelle & Condon, 2018). Reliability is a function of both task and sample, as samples with more restricted cognitive variability (e.g., because of a narrower age range), have lower variance in true scores, which thus makes up a smaller proportion variance in the observed score. Reliability was assessed via internal consistency metrics, which depend on correlations between different items (or split-halves) on the same test.

### Predictive validity

Unlike reliability, the concept of validity remains philosophically fraught, with no universally agreed definition (Gregory, 2014; Lissitz, 2009; Markus & Borsboom, 2013). There is no single test for validity, but rather multiple sources of evidence should be acquired (AERA, APA, & NCME, 2014). The first way we gathered validity evidence was to estimate how strongly the tablet assessments predict teacher-rated academic skills. As Wasserman (2018, pg. 11) notes, academic ability has "long been considered an independent criterion measure of intelligence”, and indeed many of the first cognitive tests were developed for educational decision making. The strong correlation between cognitive tests and academic performance remains one of the most robust findings in the individual differences literature (Mayer, 2011; Roth et al., 2015). These effects likely go both ways, with education also improving cognitive abilities (Ritchie & Tucker-Drob, 2018). Secondly, we estimated how strongly the tablet assessments predict a traditional standardized assessment tool (WASI-II; McCrimmon & Smith, 2013). In this case, children completed both assessments individually at our laboratory, but the tablet assessments remain fully automated requiring no researcher administration.

### Measurement invariance

Does testing children in a group alter the psychological construct being tested? For example, if children are more inattentive when performing the tests in a classroom setting, this could lead to increased measurement error and alter inter-task correlations. Multi-group confirmatory factor analysis was used to compare measurement properties of the tests when the application is used in individual or group testing (Millsap & Kim, 2018; Putnick & Bornstein, 2016). This approach requires an initial measurement model to compare across groups. Various taxonomies of cognitive abilities permeate the literature, along with different labels (e.g., executive functioning, intelligence, etc.). No consensus prevails regarding the optimal factor decomposition of cognitive task data (e.g., Rey-Mermet, Gade, Souza, Bastian, & Oberauer, 2019; Karr, Areshenkoff, Rast, Hofer, Iverson, & Garcia-Barrera, 2018). Before assessing measurement invariance, we used exploratory methods to examine the factor/component structure of the tasks in the battery.

## Methods

### Open software and data

The study protocol was approved by the University of Cambridge Psychology Research Ethics Committee (PRE.2017.102). An Open Science Framework (OSF) repository (www.osf.io/xhejz/), contains analysis scripts, and the application materials and scripts. The original study data is not publicly available due to ethical constraints, but can be shared on request. A synthetic dataset generated using the synthpop R package (Nowok, Raab, & Dibben, 2016) is openly available in the OSF repository.

The RED-App was programmed by the authors using the Unity Game Engine. One benefit of using a game engine like Unity is that the project can be exported to other platforms (e.g., Windows, Android, macOS), enhancing its general applicability. In addition, running the experiment in an online browser with a touch screen device can lead to decreased display and response recording accuracy, and relies on a consistent internet connection (Bridges, Pitiot, MacAskill, & Peirce, 2020; Pronk, Wiers, Molenkamp, & Murre, 2020). Materials have been shared for others to adapt or create new tasks within the current app framework.

### Participants

The RED study was composed of two groups. A larger school cohort, which is the focus of this paper, were recruited from primary schools, and tested as a group in their usual classrooms, during 1-h sessions. Opt-out recruitment of children from eligible class year groups was conducted. We also recruited a second, smaller cohort tested at our laboratory in Cambridge, England. This cohort completed the same tablet cognitive assessments, along with brain scans (both structural and functional magnetic resonance imaging, and resting and task-based magnetoencephalography), a parent questionnaire, and traditional standardized cognitive assessments (WASI-II).

The RED school cohort is composed of 535 children who have completed at least one assessment, from six schools, and 22 classroom groups. Schools were located in the East of England. Testing occurred between June 2018 and March 2019. Participants from the school cohort were aged from 7.29 to 9.87 years (M = 8.59 years, SD = 0.66). The RED laboratory cohort is composed of 92 children (M = 8.49 years, SD = 0.84) who completed the tablet assessments. There were no exclusion criteria for participation in the large school cohort, although data was omitted where schoolteachers believed a child could not independently complete the tasks without help from a teaching assistant. Both groups had a similar mean neighborhood deprivation (*r*_*b*_ = .031, 95% CI [– .05, .11]), measured using the England Index of Multiple Deprivation (Ministry of Housing, Communities, and Local Government, 2019). The distribution of deprivation in both cohorts is provided in the OSF repository, under supplementary figures.

Because data are drawn from a large longitudinal study, sample size was not determined from a power-analysis for the analyses presented here. We planned a sample size of 600–800 and 100 for the school and laboratory cohorts, respectively. Typical difficulties in recruiting schools applied and limited the cohort size through dropout of individual head teachers after their academy head agreed to take part, and unexpected scheduling conflicts that required schools to drop out after initially agreeing to take part. Practical constraints in testing enough participants before a cut-off date (September 2019, to allow time for a 2-year follow-up investigation), limited the size of the laboratory cohort.

### Tablet cognitive assessments

**Fig. 1.**
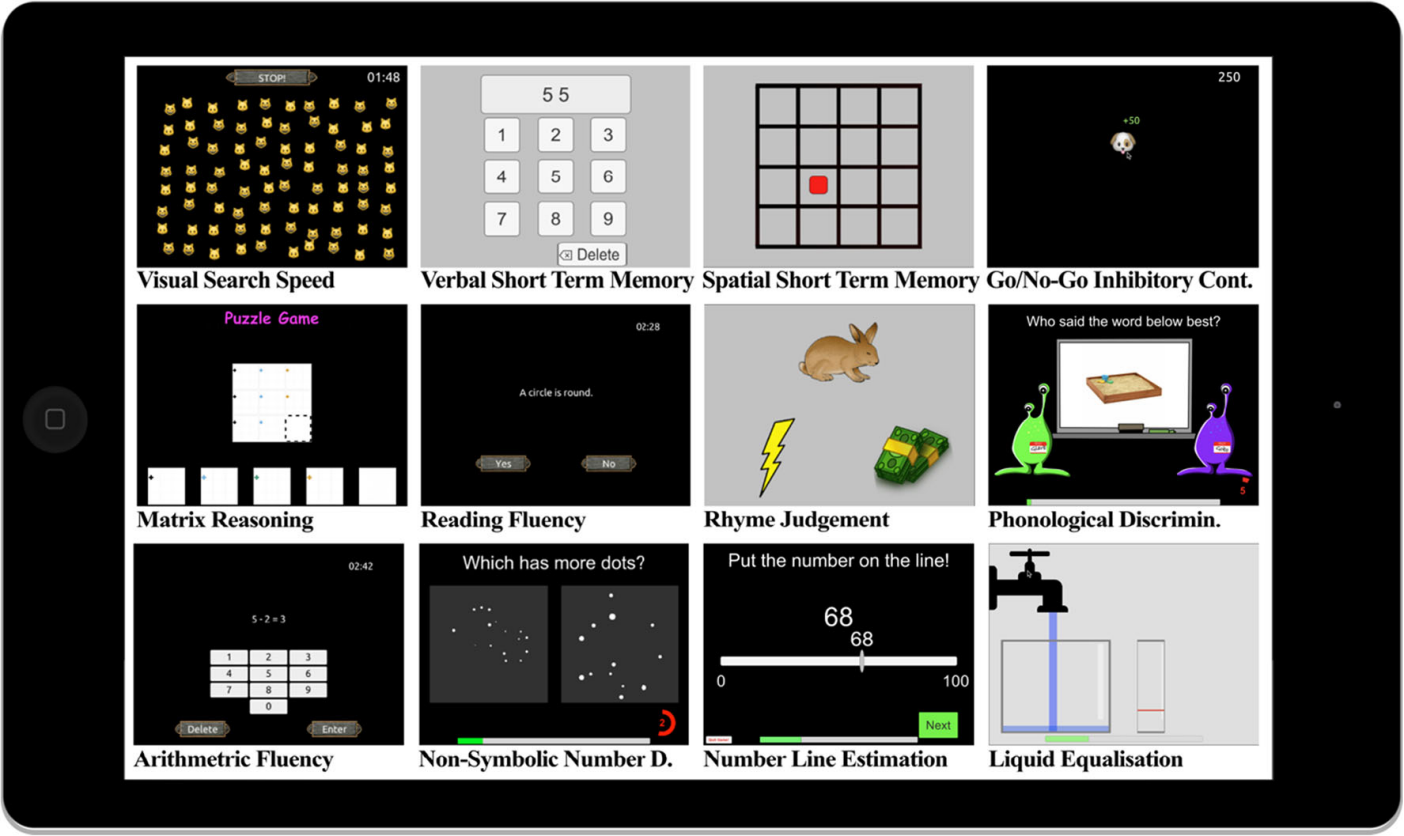
Screenshots from the novel tablet assessments

Thirty Apple iPads were used (on 9.7-inch, 1536 × 2048 resolution screens, model numbers: A1474, A1566, and A1822) for testing. After the first testing session, privacy filters were added to iPads to reduce distractions to children from nearby tablets. Children were also given large over-ear headphones to reduce external distractions and hear task instructions. We provide a brief description of each task below (see Fig. 1).

All tasks are presented in a fixed order, detailed in the accompanying GitHub repository (note the same order as presented here), so that more tasks at start of the battery were more likely to be completed. Completion rates for the two academic achievement tasks (Reading & Arithmetic fluency) and 5 “core” cognitive tasks (Visual Search Speed, Forward Digit Recall, Dot-Matrix Recall, Matrix Reasoning, & NSND) were high, but lower for other tasks (see Table 1). In designing the task order, we also aimed to alternate less subjectively enjoyable tests (e.g., matrix reasoning) with more enjoyable tests (e.g., Visual Search Speed). Some tests incorporated feedback and gamification elements which we hypothesized would increase engagement with particularly repetitive tasks, whilst other tests modeled on already standardized tasks (e.g., the memory tasks) did not. Three “quiz” rounds including questionnaires were also interspersed between tasks.

**Table 1:** Descriptive statistics for tablet cognitive assessments

Task name	*N*	Predictive validity				Reliability			Time taken		
		*r*	LB	UB	b*	ω	LB	UB	10%	50%	90%
Visual Search Speed	526	– .38	– .46	– .30	– .35	.74	.68	.78	1.59	2.18	3.30
Verbal Short-Term Memory	532	.43	.35	.51	.40				2.28	4.36	6.82
Spatial Short-Term Memory	532	.45	.37	.52	.41				1.55	3.05	5.30
G/N-G - D'	362	.18	.06	.30	.19	.53	.43	.62	1.70	4.32	4.68
G/N-G - Omission Errors	362	– .41	– .50	– .30	– .36	.71	.65	.77	1.70	4.32	4.68
G/N-G - Commission Errors	362	– .13	– .24	– .01	– .07	.60	.51	.67	1.70	4.32	4.68
Matrix Reasoning	515	.48	.40	.55	.46	.76	.73	.78	2.55	4.17	6.38
Reading Fluency	535	.63	.57	.69	.66	.87	.85	.89	3.15	3.20	3.32
Rhyme Judgement	488	.27	.18	.36	.26	.68	.59	.76	1.11	3.52	4.25
Phonological Discrimination	275	.26	.12	.39	.23	.72	.67	.78	4.73	5.32	6.54
Arithmetic Fluency	535	.55	.48	.61	.52	.89	.87	.91	3.48	3.53	3.65
Non-Symbolic Num. Discrim.	515	.45	.37	.53	.42	.85	.83	.86	3.32	4.00	4.72
Line Estimation	193	.53	.42	.63	.53	.92	.90	.94	2.20	3.22	4.44
Liquid Equalization	348	.42	.32	.51	.41	.88	.85	.90	3.02	3.53	4.21

*Note*: this table only includes data from the school cohort. Predictive validity was assessed by the linear Pearson correlation with teacher-rated academic ability, along with the lower and upper bounds of a 95% confidence interval (LB, UB). We also report the standardized regression coefficient (b*) for each task predicting academic ability whilst accounting for age and normalized neighborhood deprivation, in a multivariable regression performed separately for each task. The 10%, 50% (median) and 90% percentiles of time taken in minutes to complete each task are reported in the final three columns.

#### Visual search speed

Children are presented with an array of cartoon cat faces that are either smiling or neutral, over two rounds. In both rounds, they are asked to tap all the smiling cat faces as quickly as possible. A round ends if all smiling cats were found, the stop button is pressed, or two minutes have passed. In the first round, tapping a smiling cat produces auditory feedback (“meow!”) over the headphones, and visibly marks it with a red cross. The second round is identical but tapping a smiling cat does not leave a visual mark, requiring children to remember targets they previously clicked. Cancellation tasks feature in various large cognitive batteries (e.g., Woodcock-Johnson-II) and are thought to assess processing speed and visual attention (Dalmaijer, Van der Stigchel, Nijboer, & et al., 2015). The task was scored by calculating the 80% Winsorized (trimming the top and bottom 10% of values) mean time taken between successful cancellations in the “marked” cancellation round.

#### Verbal short-term memory

We implemented a standard digit recall memory test (e.g., Alloway, 2007). Children are presented with a series of digits (visible on-screen and spoken aloud over the headphones) in a sequence, and are asked to repeat the sequence on a virtual number pad. Sequence length begins at three digits and after getting at least four correct (out of a possible six trials) they move up a level in span length (up to a maximum of nine digits). The task was scored using the number of correct sequences performed.

#### Spatial short-term memory

We implemented a standard dot-matrix memory test (e.g., Alloway, 2007). Children are presented with an empty 4x4 matrix, and a series of red square dots light up in each box in a sequence. Children are then asked to repeat the sequence by pressing the relevant squares in each part of the matrix in the same order. The sequence length then increases using the same rules like the forward digit recall task, and is scored identically.

#### Go/no-go inhibitory control

Children are asked to quickly tap a target stimulus (a dog emoji) when it appears on the screen, and to avoid pressing the distractor (a poop emoji). The order and timings of stimulus presentation were randomized. Pressing the target stimulus elicited a barking noise, and pressing the distractor elicited a fart noise. A feedback numerical “score” was presented on the top right corner which increased 50 points for pressing a target stimulus and reduced 150 points when pressing the distractor. We scored three outcomes: the number of omission errors (failure to tap target), the number of commission errors (tapping a distractor stimulus), and the sensitivity index (d’).

#### Matrix reasoning

A two-part matrix reasoning assessment was used to measure abstract problem-solving skills. In the first part, children are presented with a series of abstract figures and are asked to select which of five drawings would appear next in the sequence. In the second part, children are presented with five abstract figures and asked to identify the figure that is different from the others. The task was scored by estimating latent ability from an item response theory analysis of each trial (detailed in the Data Analysis section).

#### Reading fluency

Children are asked to read short statements (mean character length = 30.8) and decide if the sentence is correct or incorrect as quickly as possible. The statements are designed to assess reading skill rather than knowledge, so are relatively straightforward (e.g., “A dog can fly”). The task ends after 3 min and is scored by the number of correct responses minus the number of incorrect responses.

#### Rhyme judgement

Phonological awareness was assessed with a rhyme judgement task. Each trial begins with a recorded voice stating, “does X rhyme with Y, or Z”, with visual depictions of each word being presented in line with the audio. The child responds by clicking on the best rhyming image. The presentation of the correct rhyme pair is left/right balanced across the whole set. The rhyming targets consisted of two or three syllables, and could include ‘near-rhyme’ distractors for added difficulty. All words were matched for word-frequency, age-of-acquisition and concreteness (so they could be easily identified by images) using the Kuperman, Stadthagen-Gonzalez, & Brysbaert (2013) database. Initially, the task consisted of 22 items, though following an initial analysis of the task data we reduced the number of items to six (see Results). The task was scored identically to the matrix reasoning task.

#### Phonological discrimination

A phonological discrimination task adapted from Davis et al., (2019) was used to test children’s perceptual acuity of speech. For each trial, the child hears a female-sounding “teacher” pronounce a monosyllabic noun (e.g., “fan”), which is also visually presented on a “whiteboard”. They then hear two male-sounding aliens on either side of the whiteboard consecutively repeat either the same or a different word with a single articulatory feature change (“van”). The child is asked to tap on the alien which repeated the teacher’s word most accurately. Task difficulty is modulated by altering how similar the Alien’s words sound. We selected 44 concrete word pairs and used a range of difficulties, using data and materials provided by its authors. Response accuracy was indicated by a bell with a high (correct) or low (incorrect) pitched tone after selection. The task was scored identically to the matrix reasoning task.

#### Arithmetic fluency

Children are asked to solve as many arithmetic problems as possible in 3 min, using a virtual number pad. The task ends after 3 min and is scored identically to the reading fluency test.

#### Non-symbolic number discrimination (NSND)

This task requires children to pick which of two clouds of dots is more numerous, assessing a visual number sense (Odic & Starr, 2018). Task difficulty is modulated by altering the ratio of numbers presented, as larger differences are easier to discriminate. After three simple training rounds including verbal feedback, 116 testing trials are presented and used for scoring. Six different ratios of dots are used (4, 2, 1.5, 1.3, 1.2, 1.1). Response accuracy was indicated by a bell with a high (correct response) or low (incorrect response) pitched tone after selection. Gebuis & Reynvoet (2011) script was used to generate stimuli so that the number of dots presented is minimally correlated with dot size or spatial extent. The task was scored identically to the matrix reasoning task.

#### Line estimation

Children are asked to mark points on a number-line, anchored at 0 and 100, where a given number would be located. Forty trials were presented in a fixed order. The task is scored by ranking each child on each problem by accuracy (absolute difference between target number and the line position selected), and normalizing the percentile ranks on each trial using the procedure described below. Then, the average normalized *z*-score on the 40 trials is calculated for each child and normalized again.

#### Liquid equalization

This task is an adaptation of Piaget’s equalization test (Silverman & Rose, 1982). Children are presented with two 2D empty glasses of water. They are asked to pour an amount of liquid into a left glass, that would fill the glass on the right up to a red line marking. Both the width of the glass on the right and the height of the red line change on each trial, so children have to utilize knowledge of 2D areas. On each trial a feedback “score” is given depending on the pouring accuracy. The task is scored identically to the line estimation task.

### Other assessments

Teachers completed a shortened Academic Performance Questionnaire (APQ; Bennett, Power, Eiraldi, Leff, & Blum, 2009). We selected the three items, “compared to the average student in your class, how well is the child: (1) able to read orally, (2) able to write short stories and class assignments, (3) able to perform math calculations”. The second item was added after visiting the first school, so is missing for 30 children. Teachers answered questions using a digital visual analogue scale, anchored at “well below average” to “well above average”. Sixteen teachers fully completed the questionnaire, with APQ information on 446 children in total. Mean imputation on the APQ was used for the 30 children with one missing item. Teacher’s responses across the three items were highly consistent (ω = .97, 95% CI [.94, .96]). Children in the laboratory cohort completed the WASI-II vocabulary and reading subtests. For both variables, a factor score was estimated using the ten Berge method in the *psych* R package (Grice, 2001).

### Data analysis

All analyses were conducted using R (v3.6.2), with scripts and associated files openly available (https://osf.io/xhejz/). Two approaches were taken to ensure computational reproducibility. The R package renv (Ushey, 2020) was used to manage R package dependencies, and will install the required the packages and versions used here. We also provide a docker container which captures a compatible computational environment for running the scripts (Nüst, Eddelbuettel, Bennett, Cannoodt, Clark, Daróczi, ... Petegem, 2020).

All task outcomes (apart from the Go/No-Go d’) were normalized. First, percentile ranks were estimated (ranging between 0 & 1) for scores on each test. A child who scored higher than 90% of their peers would have a percentile rank of 0.9. The percentile ranks were than mapped onto a standard normal distribution using the normal quantile function. This typical procedure for standardizing score limits the influence of univariate outliers, and maps raw scores onto an easily interpretable scale (Gregory, 2014).

#### Reliability

For tasks where all children complete an identical set of items, Revelle’s omega total (ω) was used to determine internal consistency from item-level factor analyses (McNeish, 2018). The R package *psych* (v1.8.12, Revelle, 2018) was used to estimate omega (setting number of group factors to 1), and custom code implemented non-parametric percentile bootstrapping to estimate confidence intervals with 1000 resamples. For timed tests with number (in)correct outcomes (Go/No-Go, Reading Fluency, Arithmetic Fluency), the split-half reliability was estimated also using omega. Internal consistency could not be estimated this way for the short-term memory tasks because the task ended early when participants gave consecutive incorrect responses.

For tasks with binary correct/incorrect trial outcomes (Matrix Reasoning, Rhyme Judgement, Phonological Discrimination, Non-Symbolic Number Discrimination), we also estimated reliability using item response theory (IRT). One advantage of IRT analyses is that measurement error is estimated conditional on true ability level, rather than a constant. For example, a multiple-choice mathematics test with only very simple questions may be good at discriminating between individuals with very poor mathematics ability, but ineffective at discriminating between above-average students. The mirt R package (v1.30, Chalmers, 2012) was used to fit a two-parameter (slope and difficulty) IRT model, using the Oake’s method for estimating parameters. The guessing parameter was fixed at the reciprocal of the number of choices on a given test (e.g., 25% for a four-choice test). Items with a negative discrimination parameter were removed from final models. Expected a-posteriori factor score estimation was used to calculate children’s performance on each task, using the mirt::fscores function.

#### Predictive validity

Multivariable regression was used to estimate the extent to which numerous tablet cognitive assessments can jointly explain teacher-rated academic ability, and the WASI-II scores. The main outcome is the percentage of variance that can be explained by the tablet assessments. The adjusted *R*^2^ metric was used which corrects for bias in the standard *R*^2^ (Ohtani, 2000). A bias-corrected and accelerated bootstrap 95% confidence interval for *R*^2^ was estimated using the “boot” R package (6000 resamples; Davison & Hinkley, 2019).

For predicting teacher-rated academic ability, we separately reported prediction accuracy when using the two “achievement” tests (reading and arithmetic fluency) or the other five core cognitive tests (Search Speed, Matrix Reasoning, Dot-Matrix Working Memory, Forward Digit Span & NSND). These analyses were separated as we were interested in the extent to which the Academic Achievement outcome is best explained by academic achievement tablet measures, compared to the more general cognitive measures. All seven measures were also combined to estimate the overall variance explained by tablet tasks. Due to low completion rates, we did not include supplementary tests in these analyses because the high rate of non-completion would reduce the sample size for the analyses. Missing data were imputed for participants with only one missing tablet assessment out of the seven. The classification and regression trees imputation method from the MICE R package was used (Van Buuren & Groothuis-Oudshoorn, 2011).

The number of children each teacher rated varied from 10 to 32, with a mean of 26.24. If teachers systematically over- or under- estimated children's performance, then observations cannot be treated as statistically independent. To account for potential non-independence, the R package lme4 was used to fit random-effect models with restricted maximum likelihood estimation (Bates, Mächler, Bolker, & Walker, 2015). The intraclass correlation coefficient (ICC), the proportion of variance explained by the random effects, is reported for each model. Confidence intervals were calculated using lme4’s percentile bootstrap method. We used the same imputed data as described above.

The same approach was taken to estimate prediction accuracy for WASI-II. Note that because children were tested in individual sessions, more tasks were completed overall and there was fewer missing data on some tasks. Therefore, in the regression model all task outcomes in Table 1 were used apart from Line Estimation, Rhyme Judgement and Liquid Equalization which has the lowest completion rates (< 58%). For the remaining 11 task outcomes, the same imputation method was used, but children were excluded if tablet cognitive data was missing for more than three variables.

#### Measurement invariance

We assessed measurement invariance using standard psychometric procedures employed in the R functions Lavaan::cfa (v0.6-5, Rosseel, 2012) and semTools::measurementInvariance (v0.5-2, Jorgensen, Pornprasertmanit, Schoemann, & Rosseel, 2018), presented in Table 2. This implements a standard multi-group confirmatory factor analysis approach for checking measurement invariance across the school and laboratory-tested cohorts. It works by running confirmatory factor analyses in both groups, and over a series of model changes, it constrains additional parameters (i.e., loadings, intercepts, residual and means) to be equivalent across groups. Constrained models are compared against the unconstrained “configural model”, to compare model fit indices. Multiple model fit indices are presented, including Chi-square (χ^2^) and its corresponding *p* value, comparative fit index (CFI), root mean square error of approximation (RMSEA), and Akaike Information Criterion (AIC).

**Table 2:** Assessment of measurement invariance across the group-tested (*N* = 92, school) and individually tested (*N* = 535, laboratory) cohorts

Models	χ^2^			Comparative Fit Index (CFI)				Root mean square error of approximation				AIC
	*χ*^2^	*df*	*p*	CFI	Δ	LB	UB	RMSEA	Δ	LB	UB	
1. Configural	63.4	28		.971				.0635				11012
2. Loadings	73.8	34	.107	.967	– 0.004	– .024	.001	.0611	– 0.002	– .010	.010	11010
3. Intercepts	98.3	40	<.001	.952	– 0.015	– .038	– .005	.0682	0.007	– .004	.018	11023
4. Residuals	107	47	.273	.951	– 0.001	– .023	.001	.0638	– 0.004	– .008	.005	11017
5. Means	113	48	.015	.947	– 0.004	– .014	.001	.0657	0.002	– .001	.006	11021

*Note:* The lower (LB) and upper bounds (UB) of the 95% confidence interval for changes (Δ) in CFI & RSMEA are reported, calculated using non-parametric bootstrap resampling (3000 repeats). Random sampling was performed within each group separately for each iteration.

Conventionally, changes in CFI of less than – 0.01, or changes in RMSEA of greater than 0.01, are interpreted as evidence of measurement invariance (Putnick & Bornstein, 2016). The model with the lowest AIC is said to be best. To estimate uncertainty in CFI and RSMEA from sampling error, we estimated percentile bootstrap confidence intervals (3000 repetitions).

Because this requires a measurement model to compare across groups, the task factor structure was explored in the school cohort first. Parallel analysis and Velicher’s minimum average partial correlation (MAP) method were used to determine the optimal number of components/factors to explain variance in task scores, using the *psych* R package. MAP finds the number of components that minimize the average squared residual correlations. Parallel analysis compares the variance explained by each factor/component, to the variance explained when the data has been randomly permutated (repeated 1000 times). Factors/components are kept until they explain equal to or less variance than in the permutated datasets.

## Results

The time taken to complete each task is reported in Table 1. For most tasks, 90% of children completed them in under 5 min, though some tasks such as matrix reasoning and verbal/visual short-term memory took slightly longer.

### Reliability – omega total (ω)

Descriptive information on each task including internal consistency, are presented in Table 1. Most tasks have good reliability (between 68% and 92%), except for two of the Go/No-Go outcomes.

### Reliability - item response theory (IRT)

The two-parameter IRT model failed to converge (within < 30,000 iterations) for the NSND task. The larger number of trials (116) in NSND requires estimating many parameters (232), perhaps requiring a greater sample size. Instead for NSND, a simpler Rasch model was used estimating only a difficulty parameter.

**Fig. 2.**
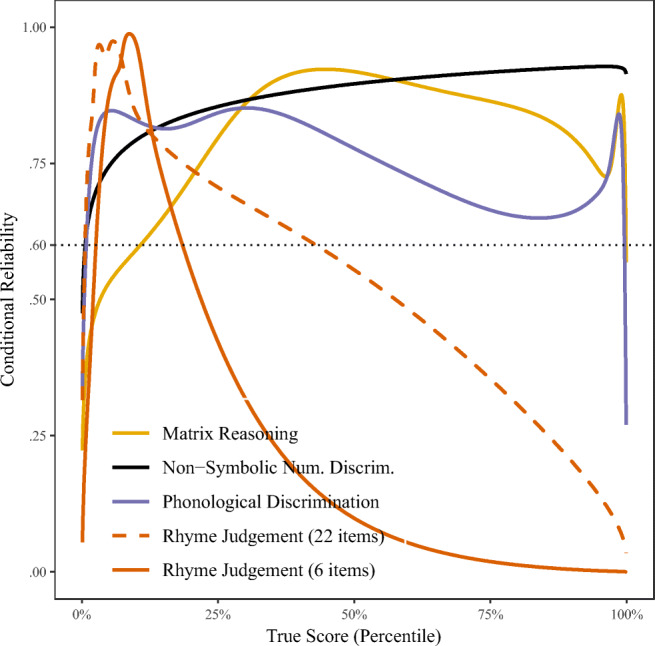
Test reliability function, modeling measurement error as a function of latent ability, rather than a constant

A Test Reliability Function (Fig. 2) for each task was computed using the mirt package, where the *x*-axis presents latent ability on a uniform scale. Despite adequate omega reliability, the IRT analysis demonstrates that the rhyme judgement task is largely poor at discriminating between children above the 20% ability percentile. Indeed, a strong ceiling effect is evident for this task, as median participant accuracy was 95.4%. Other studies report that ability to judge rhymes is at ceiling by the age of 5 years (Stanovich, Cunningham, & Cramer, 1984,

Sumner, 2018), which we did not overcome by employing a greater number of syllables and near-rhyme distractors. This illustrates the value of IRT modeling, as the high reliability and validity metrics in Table 1 mask these limitations of the test.

Our subjective experience with the assessment overlapped with the above analysis of the rhyme judgement task during data collection. Consequently, we added the phonological discrimination test mid-way through data collection, to provide an additional, more sensitive test of phonological skills. We then reduced the number of trials to six in the rhyme judgement task. As a result, both tests were not fully completed by the whole sample. The other three assessments demonstrated adequate reliability across ability levels, though the Matrix Reasoning task would benefit from additional easier items for this age-group.

### Predictive validity: Academic ability

**Fig. 3.**
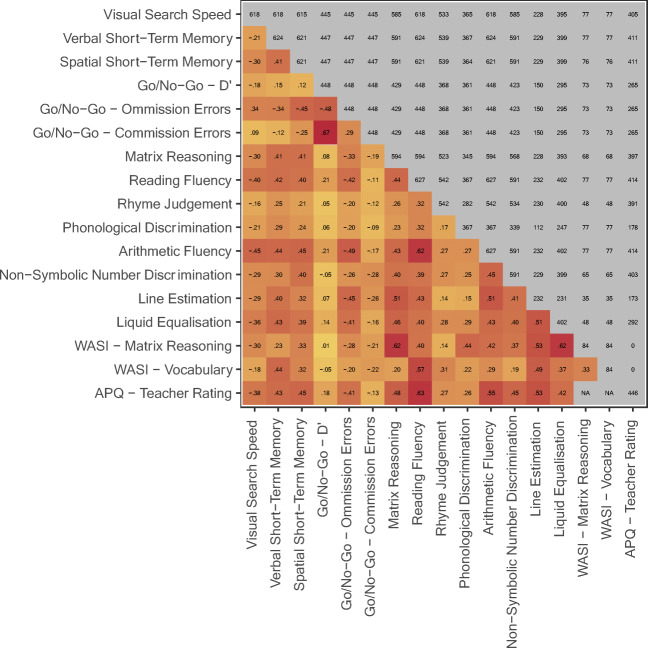
Pearson correlation matrix of primary outcomes, including all RED participants from both school and laboratory cohorts

Pairwise correlations with academic ability for all tests are reported in Table 1 and Fig. 3. We estimated how much variance in teacher-rated academic ability, measured with the APQ, could be explained by combining tablet assessments using multivariable regression.

The reading and arithmetic fluency tests alone predicted 44.9% of variance (_adj_*R*^2^ = 44.9, 95% CI [.38, .51], *df* = 411). The five main cognitive outcomes (Search Speed, Matrix Reasoning, Dot-Matrix Working Memory, Forward Digit Span & NSND) predicted 39.5% of variance (_adj_*R*^2^ = 39.5, 95% CI [.33, .47], *df* = 404). Combining all seven tablet assessments can explain just over half the variance in teacher-rated academic ability (_adj_*R*^2^ = 51.0, 95% CI [.44, .57], *df* = 402).

Because teachers provided APQ ratings for multiple children, this may violate the independence of errors assumption. Therefore, we fitted a random intercept model with the seven tasks described above as fixed effects. The random intercept accounts for mean differences in teacher ratings between classrooms, and explains 6% of variance in outcomes (σ^2^ = .066, 95% CI [.02, .14], ICC = .060). Note that in the context of mixed linear modeling the ICC is not indicative of reliability. Importantly, the tablet assessments still explained just over half the variance in teacher-rated academic ability (*R*^2^ = .55). Both fixed and random effects could explain 61% of variance in teacher ratings. Potentially there is a small tendency for teachers to over- or under-estimate ability, but the cognitive tests remain strong predictors of ability when accounting for this.

### Predictive validity: WASI-II

Children in the smaller laboratory cohort completed both the tablet tasks and the WASI-II, a standardized cognitive test. We used multivariable regression again to predict the WASI-II factor scores. Due to the higher rates of task completion in the laboratory cohort compared to the school cohort, the three Go/No-Go outcomes and phonological discrimination tasks were also added to the linear models. The 11 task outcomes combined predicted 42.4% of variance in WASI-II factor scores (_adj_*R*^2^ = 42.4, 95% CI [.31, .59], *df* = 62). This effect is large given the low correlation between WASI-II tasks, which resultingly have a low estimated reliability (omega total = .49).

### Measurement invariance: Task factor structure

Before running measurement invariance analyses, it is important to establish the latent variable structure of the tasks. We used principal component analysis and exploratory factor analysis, using data from the school cohort only. Data from the seven tasks with the highest completion rates across the two samples were used, including: Visual Search Speed, Matrix Reasoning, Dot-Matrix Working Memory, Forward Digit Span, NSND and Arithmetic and Reading Fluency).

**Fig. 4.**
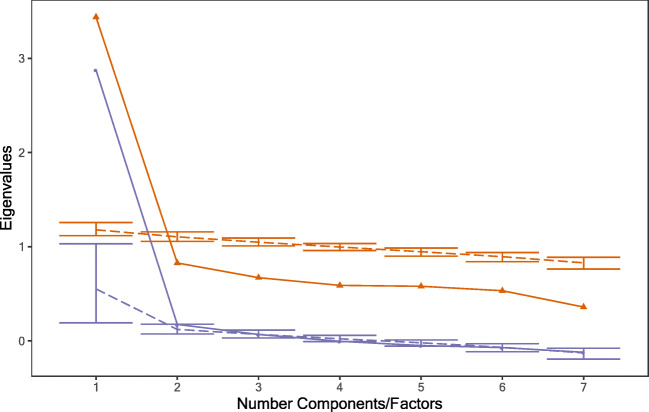
Parallel analysis for all tasks, using data from school cohort only. Note. The bottom purple line represents successive factor analysis eigenvalues, and the top orange line for principal components. The dashed lines represent eigenvalues taken from resampled datasets.

The parallel analysis (see Fig. 4) suggested that a single factor or component best explained task score variance, as extracting additional components did not explain more variance than would be expected in randomly permutated datasets. A single factor solution also minimized the MAP (0.034).

### Measurement invariance

As the previous section suggested a single-factor solution, we fitted a multigroup confirmatory factor analysis (CFA) model with a single latent cognitive factor on same seven tasks, in school and laboratory RED cohorts. Measurement invariance analyses test whether model fit is worsened by constricting loading and intercepts to be equivalent across the two groups. Results are also presented in Table 2.

First, we fitted a configural model allowing all parameters to be freely estimated in each group. In Model 2, task loadings on the latent factor are constrained to be equivalent across the two groups. This did not significantly reduce model fit (*p* = .107), and only marginally reduced comparative model fit (ΔCFI = – .004) and root mean square error of approximation (ΔRMSEA = – .002, 95% CI [– .008, .005]) measurement fit indices. The AIC also suggests that Model 2 is the preferred model. This suggests that tasks measure latent ability equally well across the two groups. The third model constrains the intercepts of task performance on latent ability; the expected task scores when latent ability is zero. This tests whether group differences in task performance cannot be explained by latent ability alone. A significant (*p* < .001) decrease in model fit is observed, with a moderate reduction in CFI (ΔCFI = – .015, 95% CI [– .038, – .005]), although the RSMEA did not increase above the conventional 0.1 threshold (ΔRMSEA = .007, 95% CI [– .004, .018]). The fourth model constrained the item residuals, the remaining variance in each item after partialing out latent ability. This did not change the CFI (ΔCFI = – .001, 95% CI [– .023, .001]), or RMSEA more than the threshold amounts (ΔRMSEA = – .004, 95% CI [– .008, .005]). The final model constrained the latent ability means to be equivalent. Again, this did not change the CFI (ΔCFI = – .004, 95% CI [– .014, .001]), or RMSEA more than the threshold amounts (ΔRMSEA = .002, 95% CI [– .001, .006]). The Chi-square test does suggest that a significant reduction in model fit occurred (χ^2^ = 113, *p* = .015).

While there is evidence for metric invariance, that overall the tasks measure the latent g-factor equally well across the groups, there may other important measurement differences. Average performance (latent ability) and performance on individual tasks (task intercepts) appear to be invariant. Inspection of Model 2 (Table 2) intercepts suggests that the lab group performed on average better on matrix reasoning (lab group intercept = .381, Bonferroni corrected *p* = .005). There was also a non-significant trend for higher performance in digit span (lab group intercept = .257, Bonferroni corrected *p* = .053). Potentially, these tasks were more negatively impacted by distractions in the classroom.

Ultimately, these results do not show that raw scores on lab and individual testing can be directly compared. The relatively wide confidence intervals suggest more evidence is needed to draw robust conclusions. Crucially, however, there is also no evidence that scores in either cohort are more informative than the other, with no evidence of different task loadings across groups. Indeed, estimated internal consistency from the seven tasks in the laboratory (omega total = .78, 95% CI [.70, .84]) and school cohorts (omega total = .82, 95% CI [.80, .84]), are comparable.

## Discussion

We found that 7 to 9-year-olds can quickly and reliably complete cognitive assessments using an automated tablet application - requiring minimal researcher assistance. Ninety percent of the sample can complete the seven core tasks in under 34 min (Table 1). Reliability was high for most assessments, around 70–90%. These assessments had strong predictive validity, explaining over half the variance in teacher’s ratings of academic ability. This compares favorably to a meta-analytic estimate that standard intelligence tests predict just over 19% of variance in school *grades* (Roth et al., 2015). Some individual tasks had low reliability and predictive validity, and could be refined or replaced in future applications. For example, the item response theory analysis found that the rhyme judgement task was too easy for most children. Two outcomes from the Go/No-Go task had low reliability (53% and 60%), though this is not uncommon for inhibitory control tasks (Enkavi et al., 2019; Hedge, Powell, & Sumner, 2018; Rouder, Kumar, & Haaf, 2019). In a smaller laboratory group who completed the tablet assessments individually, tablet performance explained 43% variance in a traditional cognitive ability assessment (WASI-II).

Analyses of measurement invariance do not show that the tablet assessments are less reliable or predictive of latent ability when used as group tests in schools, compared to when assessments are completed individually. However, there were some small differences. Differing task intercepts indicated that the lab sample performed better on the matrix reasoning task, and that the two samples had different mean latent ability levels. Potentially, the contrasting sampling procedures between the two samples could explain differences, though it is uncertain why this would impact performance on an individual task. Potentially, the more distracting school environment may decrease performance on specific tasks that require more attention, such as matrix reasoning.

We expect our results can be replicated within certain boundary conditions. It is likely that using different tasks, or similar tasks with different stimuli or procedures will affect the individual reliabilities, inter-task correlations & mean performance. For example, changes in stimuli sets used in non-symbolic number discrimination tasks can have strong impacts on measured performance (DeWind & Brannon, 2016; Smets, Sasanguie, Szücs, & Reynvoet, 2015). However, regardless of the specific tasks employed, single factors extracted from broad task batteries (“g-factors”) tend to converge on very similar estimates of cognitive ability. Indeed, Spearman termed this phenomenon “the indifference of the indicator” (Johnson, Bouchard, Krueger, McGue, & Gottesman, 2004; Thorndike, 1987; Vernon, 1989). Additionally, g-factors have been identified across a wide range of populations (Warne & Burningham, 2019). Therefore, we expect general cognitive ability estimates derived from large, heterogenous batteries of tasks to be similar across studies, even when using somewhat different tasks.

Despite the success of our measurement tool, group testing presents additional, somewhat unique, challenges. Making sure that tasks are intuitive and easy to use without help is essential.

Although some of the tasks presented here included accuracy feedback and points systems, the current evidence is highly mixed regarding its effects on engagement (Attali & Arieli-Attali, 2015; Ling, Attali, Finn, & Stone, 2017) and accuracy (Beckmann, Beckmann, & Elliott, 2009; Betz, 1977; Delgado & Prieto, 2003). More broadly, the gamification of cognitive tests has been suggested to improve engagement and data quality, but the existing evidence base is limited (Dockterman et al., 2020; Lumsden, Edwards, Lawrence, Coyle, & Munafò, 2016). We recommend extensive piloting under observation during the development of any self-administered tasks. The results from these assessments may not generalize if the testing environment is highly chaotic. Distractions can be minimized by using over-ear headphones and privacy screen filters on the tablet screen. Adding secondary tests or games at the end of a battery can keep children who finished early occupied, and avoid distracting others.

By including questionnaires in the application, children’s environments and mental health can also be measured, allowing researchers to quickly generate evidence that is of current policy interest, for example on the effects of environmental exposures (e.g., Bignardi et al., 2020; Dalmaijer, Bignardi, Anwyl-Irvine, Smith, Siugzdaite, Uh, ... Astle 2019). One relatively unexplored application of group-based testing with tablets is as a cost-effective “screener” for the early detection of cognitive difficulties. This could help recruitment of children with particular cognitive profiles for subsequent studies, or to identify children who might find learning more difficult in a conventional classroom setting, which would permit subsequent in-depth assessment and earlier targeted interventions (Gaab, 2019). However, screeners require higher evidence of reliability and practical utility. For example, even an “excellent” reliability of 90% would correspond to a 95% confidence interval of ± 9.3 points (assuming an IQ-like scale with a standard deviation of 15; Revelle & Condon, 2018). Further improvements to reliability could be possible by utilizing more sophisticated modeling techniques (Farrell & Lewandowsky, 2018; Haines et al., 2020), or adaptive testing procedures (Harrison, Collins, & Müllensiefen, 2017).

An alternative, promising approach to the one outlined here is to utilize remote online testing, and developmental researchers are increasingly exploring this option due to the COVID-19 epidemic disrupting laboratory research (Dillon, 2020). As popular online participant recruitment services do not include under 18-year-olds, researchers rely on slower, traditional recruitment approaches. As with other convenience samples, representativeness is an issue (McCredie & Morey, 2019). One innovative approach is to utilize online digital educational content, for example *Math Garden* is a popular educational tool containing multiple games for children to practice math problems (Brinkhuis, Cordes, & Hofman, 2020). Data gathered using these applications can produce large and detailed longitudinal datasets, which can be used to test learning theories (e.g., Hofman et al., 2018).

## Conclusions

The importance of measurement is often overlooked in psychological research, which can be seen in the proliferation of “Questionable Measurement Practices” (Flake & Fried, 2019). More efficient measurement practices, such as described in this paper, can also advance the field by allowing larger samples of cognitive data to be rapidly acquired, which are required for making robust inferences. Here, we outline a methodology for rapidly acquiring large datasets of cognitive data in school-aged children. With 2–4 researchers overseeing data collection, data on up to 30 children can be acquired in an hour, or 150 children in a school day. Cognitive data collected in this way has good reliability and validity evidence.

### Author Note

The Resilience in Education and Development (RED) Study is supported by grant TWCF0159 from the Templeton World Charity Foundation, and by the UK Medical Research Council. An earlier version of this article has been shared on OSF Preprints (https://osf.io/aw6c5/), and portions of the findings have been presented as posters at the 2019 British Neuroscience Association (BNA) and 2019 British Association for Cognitive Neuroscience (BACN) conference. We have no conflicts of interest to disclose.
